# 

**Published:** 1879-07

**Authors:** 


### Table of Contents.

### ORIGINAL COMMUNICATIONS.

Canthotomy for the Relief of Blepharospasm. *By Chas. W. Miles, M.D., Jordan, Ky* ........ 193

Resuscitation from Chloroform Syncope. *By E. F. Starr, M.D., Nacoochee, Ga* ........ 195

### REPORTS OF SOCIETIES.

Atlanta Academy of Medicine ........ 198, 200
The American Association for the Cure of Inebriates ........ 203
Academy of Medicine—Section on Theory and Practice ........ 210

### SELECTIONS.

Malignancy in Tumors ........ 212
Necrosis ........ 222
Chloric Acid ........ 231
Alcoholic Dressing of Wounds, as a Phophylactic against Erysipelas, 234
A Summary of Ferrier's Localizations ........ 237

### EDITORIAL.

The Mode of Securing Medical Education ........ 242
Malpractice ........ 245

### BIBLIOGRAPHICAL.

Elements of Modern Chemistry ........ 246
Diseases of the Intestines and Peritoneum ........ 247
Transactions of the American Gynæcological Society for 1878 ........ 247
Posologicol Table ........ 248
A Treatise on the Horse, and His Diseases ........ 248
American Health Primers ........ 248
Meteorological Report for June ........ 249

### EXCERPTA.

Habitual Constipation ........ 250
The Name "Allopath" ........ 251
Sweat Producing Agents ........ 252
Remarkable Sympathetic Disturbance ........ 255
Lactopeptine ........ 255
Poisoning by Carbolic Acid ........ 256
The Metric System ........ 256

